# Supplementary material for: Optical Detuning Strategies for Shielded Loop Resonators
Source: Magn Reson Med. 2026 Aug 3;96(6):3011–20. doi: 10.1002/mrm.70556 (PMC13619850; doi:10.1002/mrm.70556)
Supplement: Supplementary file 1 [file MRM-96-3011-s001.docx]

SUPPORTING INFORMATION

Supporting Information Figure S1: (A) A detailed ADS simulation model used for the simulation of S11 curves (in this specific case of the PD+PIN network). The models vary depending on loop parameters, detuning method and components. Loop *L* and *R* were measured with an impedance analyzer, the transmission line characteristics taken from the datasheet (SUCOFORM_47_CU_LSFH). (B) displays the model in cases, where the detuning network is located across the gap (Cross-diodes or D-PT, this example: Cross-diodes). (C) Composition of measured unloaded and loaded S11 curves for each detuning circuit, contrasted with simulation results.

Supporting Information Figure S2: Photos of measurement setup on patient table. A) Open setup displaying interfaces, connections and fibers leading to the headrest. B) Setup covered underneath patient board. C) Side view of volunteer lying on the board, parts of the setup still visible.

Supporting Information Figure S3: Comparison of individual channels and combined image (2D-FLASH, TR/TE=100/10ms, α=25°, FOV=250×250mm^2^, ΔV=1×1×3mm^3^, *N*_averages_=5). (A) indicates the placement of the individual coils on the phantom with respect to the measurement plane. (B) presents images acquired by one channel each while the others are detuned, channel 1 and 3 are rescaled to account for the difference in proximity to the measurement plane. (C) displays a phantom measurement with 4 coils (adaptive combination).

Supporting Information Figure S4: Comparison of SNR of galvanic and optical detuning with the PD+PIN method (2D-FLASH, TR/TE=100/10ms, α=25°, FOV=250×250mm^2^, ΔV=2×2×5mm^3^). Galvanic detuning is shown on the left side, PD+PIN detuning on the right side. The top row presents transverse, the bottom row sagittal imaging planes recorded with a loop coil introduced in [29].

## PD output biasing the PIN diode

In the PD+PIN design presented in this work detuning does not correspond to full activation (“switching on”) of the PIN diode. Instead, the photocurrent and voltage of the PD under irradiation changes the bias of the PIN diode, altering its resistance to the AC measurement signal. Supporting Information Tables 1 and 2 display bench test results to illustrate the process.

Supporting Information Table 1: *V*_OC_ and *I*_SC_ of the OSRAM SFH 203 P photodiode at different optical powers *P*_Opt_ (852 nm).

| ***P*_Opt_** [mW] | 0.0 | 1.5 | 2.5 | 5.0 | 10 | 20 | 50 |
| --- | --- | --- | --- | --- | --- | --- | --- |
| ***V*_OC_** [V] | 0.27 | 0.48 | 0.50 | 0.52 | 0.55 | 0.57 | 0.60 |
| ***I*_SC_** [mA] | 0.00 | 0.35 | 0.57 | 1.32 | 3.15 | 7.48 | 17.5 |

For the PIN diode (MACOM MA4P7470F-1072T), the datasheet lists a maximum forward voltage of *V*_F_ = 1.0 V when measured with a forward current of *I*_F_ = 100 mA. In a real diode curve, however, the diode is not suddenly switched at this maximum forward voltage, but gradually switched or activated. With a DC source delivering 3 mA input current to the PIN diode (similar to the PD’s *I*_SC_ at *P*_Opt_ = 10 mW) the voltage is varied. At 0.739 V, 2.7 mA (90% of the input current) passes the PIN diode, measured with a multimeter. While the activation or switching of the diode is therefore not a sudden shift, but a gradual change, the voltage supplied by the PD is significantly lower (see Supporting Information Table 1). The resulting resistance of the PD+PIN setup for the AC measurement signal is displayed in Supporting Information Table 2.

Supporting Information Table 2: Series resistance *R*_S_ of the PD+PIN setup (including the *L*=33 µH choke inductances separating PD and PIN diode), measured for varying optical powers *P*_Opt_ with an impedance analyzer at a frequency of 123.2 MHz.

| ***P*_Opt_** [mW] | 0.0 | 1.0 | 2.0 | 5.0 | 10 | 20 | 50 |
| --- | --- | --- | --- | --- | --- | --- | --- |
| ***R*_S_** (@ 123.2 MHz) [Ω] | 512 | 316 | 188 | 98.5 | 60.1 | 41.5 | 27.3 |

A previous work by Saniour et al. (2017) [14] used two PDs in series, which was probably needed to reach the voltage to fully switch the PIN diode. In our case, the photocurrent of the PD changed the bias condition of the PIN diode which was sufficient for detuning.

To isolate the measurement signal (AC) from the detuning signal (DC), the PD+PIN design features *L* = 33 µH choke inductors (Coilcraft 1812CS-333). Inspite of their higher resistance of *R* = 13.5±0.1 Ω they are preferable over lower inductance values, e. g. *L* = 1.2 µH inductors (Coilcraft 1812CS-122) which feature only *R* = 1.05±0.01 Ω. When replacing the *L* = 33 µH inductors with *L* = 1.2 µH inductors, bench tests showed no notable difference (≈1%) in isolation between tuned and detuned state (Δ_S21_), yet the unloaded *Q* deteriorates by about 25 % (see Supporting Information Figure S5B). This shows that high isolation between AC and DC current paths is vital to preserve *Q*.

  Supporting Information Figure S5: S-parameter comparison for the SLR with various detuning networks. A) Single- vs. double-PIN diode detuning implemented on the same SLR. It also displays a 6 dB drop in peak S21 when a coil is terminated with 50 $\Omega$. B) PD+PIN detuning network with RF choke inductors of *L* = 33 µH (left column) and *L* = 1.2 µH (right column). No significant difference in ΔS_21_ was observed, while the network with lower inductor resulted in a lower Q of the SLR. C) Performance comparison of passive detuning network using crossed-diodes positioned either across the gap or between inner and outer conductor.

Supporting Information Figure S6: Body coil images with surface coil present, detuned with the PD+PIN method at *P*_opt_ = 10 mW (2D-FLASH, TR/TE=100/10ms, FOV=200×200mm^2^, ΔV=0.8×0.8×3mm^3^). Displayed is signal intensity at several flip angles, illustrating detuning at varying RF pulse amplitudes. Depending on flip angle, the mean signal of a circular ROI close to the detuned surface coil (orange circle in α = 40° measurement) is 61.4% – 85.6% that of an ROI in the center of the phantom (red circle in α = 40° measurement), as compared to 79.0% in a reference measurement without surface coil (not depicted).

Supporting Information Figure S7: Photo of S21 measurement setup. The investigated coil is fixed on a phantom in the center of the image. Above the coil, a double-loop-probe is fixed at a distance of around 3 cm and connected to a VNA. The port of the coil is connected to a Tee-bias circuit to deliver a galvanic detuning signal.

Supporting Information Figure S8: Oscilloscope measurements of PD+PIN tuning and detuning speed with an optical switch (NanoSpeed 1X2, Agiltron, Woburn, MA, USA). An RF signal is coupled from a sniffer loop to a PD+PIN coil, which is connected to Channel 3 of the oscilloscope. A trigger signal is sent to the switch, the output of the switch is split between a simple PD as detector (connected to Channel 2) and the PD+PIN detuning network. At the top is a full cycle of switching between tuned and detuned state. In the middle is a detailed view of the tuning process, which takes about 19.3 µs. At the bottom is a detailed view of detuning, which takes about 1.7 µs and is faster than the switch-off of the direct switch output.
